# Energy Efficient Data Transmission for Sensors with Wireless Charging

**DOI:** 10.3390/s18020511

**Published:** 2018-02-08

**Authors:** Xiaolin Fang, Junzhou Luo, Weiwei Wu, Hong Gao

**Affiliations:** 1 School of Computer Science and Engineering, Southeast University, Nanjing 211189, China; jluo@seu.edu.cn (J.L.); weiweiwu@seu.edu.cn (W.W.); 2 School of Computer Science and Technology, Harbin Institute of Technology, Harbin 150001, China; honggao@hit.edu.cn

**Keywords:** wireless charging, thermal effect, data transmission

## Abstract

This paper studies the problem of maximizing the energy utilization for data transmission in sensors with periodical wireless charging process while taking into account the thermal effect. Two classes of problems are analyzed: one is the case that wireless charging can process for only a limited period of time, and the other is the case that wireless charging can process for a long enough time. Algorithms are proposed to solve the problems and analysis of these algorithms are also provided. For the first problem, three subproblems are studied, and, for the general problem, we give an algorithm that can derive a performance bound of $\left(1-\frac{1}{2m}\right)\left(OPT-E\right)$ compared to an optimal solution. In addition, for the second problem, we provide an algorithm with $\frac{2m}{2m-1}OPT+1$ performance bound for the general problem. Simulations confirm the analysis of the algorithms.

## 1. Introduction

Wireless charging has an extensive future in many areas [1,2,3,4,5,6,7], where the devices such as implanted sensors are expected to work for a long time, and it is inconvenient to replace batteries. Therefore, wireless charging technology is required for long-term monitoring. As stated in [5], wireless power transfer enables various devices to keep working by powering or recharging them safely, efficiently, and over a distance. The benefits of using this technology range from enabling implanted architectures that could not otherwise be possible or practical, in order to improve the productivity and safety of the sensors.

However, the wireless charging process can result in a thermal effect, which is a critical concern in biosensors [8,9,10,11]. It has been stated that some organs are very sensitive to any temperature increase and prone to thermal damage because of a lack of blood flow to them [11,12], and even a slight thermal rise would cause damage. Therefore, the sensors should not receive wireless energy all the time, and the wireless charging process shoud be intermittent so that the thermal effects can be eliminated after charging for a while. As the human body has its own cooling process, we consider a periodical wireless charging process where in each period the sensors will receive wireless energy first, and, for the rest of the period, it is used to eliminate the thermal effect in this paper.

Due to the capacity constraint of the batteries, the sensors can store no more energy than their capacity, thus the over received energy will be wasted. If a battery is almost full, then, after a period of wireless charging, the battery gets full, but some energy may be wasted because there is not enough capacity to store energy in the battery, that is, energy overflowing occurs. In this case, it does not take full advantage of the whole period of wireless charging. This paper formalizes a problem to improve the energy utilization rate so as to transmit as much data as possible in the periodical wireless charging situation. The contributions of this paper are summarized as follows:

- To the best of our knowledge, this is the first work to study data transmission in sensors taking into account the thermal effects in wireless charging and uses a periodical charging period to eliminate the thermal effect.
- This paper considers the problem where the wireless charging continues for a limited period of time. Algorithms are provided and the performance bounds of the algorithms are also analyzed.
- This paper also considers the problem where the wireless charging continues for a long enough time. Algorithms are provided and the performance bounds of the algorithms are also analyzed.
- The simulations are conducted to evaluate and confirm the performance of the provided algorithms.

The rest of this paper will first introduce the motivation and the problem formulation, and then study the problem with different conditions. After that, the simulation results are presented, then the related work follows, and finally it concludes this paper.

## 2. Motivation and Problem Statement

### 2.1. Intermittent Wireless Charging

Previous works have shown that wireless electromagnetic energy transfer through induction, radio frequency waves, or resonant evanescent coupling results in thermal effects on tissues [8,11,12,13,14,15,16,17]. The temperature of a wireless power receiver will rise as the energy is being transferred. It is stated that exposure to SAR (Specific Absorption Rate) of 8 W/kg for 15 min has shown to result in severe tissue damage [11]. Organs like lenses are especially sensitive to any temperature increase and prone to thermal damage because of a lack of blood flow to them [12], and even a slight thermal rise would cause damage. Thus, we must consider the thermal issues in human tissues during wireless charging. Four cooling mechanisms of radiation, conduction, convection and perspiration can cool human body, and the blood flow can also take heat from hot areas to cool areas so as to keep temperature balanced [18,19]. In a word, the body can cool down itself and keep the temperature within certain boundaries automatically after a certain time.

**Figure 1:**
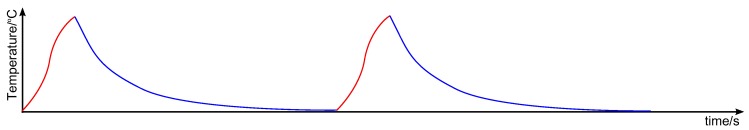
Periodical wireless charging process.

Therefore, we use a periodical wireless charging mechanism to eliminate the thermal influence. In the beginning of each period, the sensors receive wireless energy, thus the temperature increases as shown in the red curve in Figure 1. When the temperature increases to a certain degree, it should stop wireless charging to wait the temperature to reduce to a safe degree, as shown in the blue curve in Figure 1. Usually, the cooling process is much slower than that of the temperature increasing process; thus, the duration of the blue curve is usually much longer than that of the red curve.

### 2.2. Energy Waste

**Figure 2:**
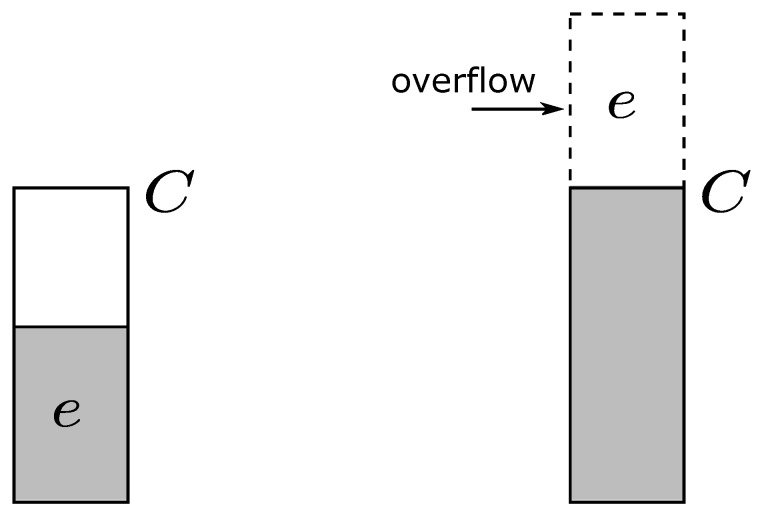
Illustration of energy overflowing.

Energy is a rare resource; therefore, it requires an efficient way to avoid wasting energy in low power biosensors. It includes two kinds of energy wastes. The first is that it does not receive the wireless energy efficiently, and the second is that it does not fully utilize the received energy. In each charging period, sensors need to receive as much energy as possible. If the battery is almost full, then the energy may overflow in the next period of wireless charging. The energy overflowing is illustrated in Figure 2. Let the energy that can be received in each period be *C*, and the battery can store at most *C* units of energy. Let the energy left in the battery be *e*; then, *e* units of energy will overflow in the next period as shown in the right part in Figure 2. Energy overflowing is one type of energy waste in this paper.

**Figure 3:**
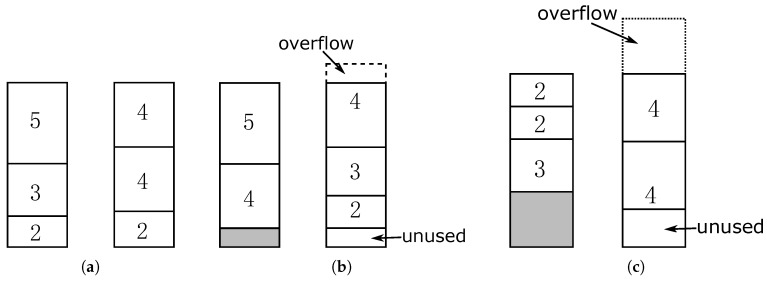
Different transmission orders result in different energy waste. (**a**) Order of 5,3,2,4,4,2; (**b**) Order of 5,4,4,3,2,2; (**c**) Order of 2,2,3,4,4,5.

Besides the energy overflowing, unused energy is another type of energy waste. Figure 3 demonstrates an example of the energy waste. Given *n* packets to be transmitted, packet *i* is of size $s_{i}$, which denotes that it will cost $s_{i}$ units of energy to transmit this packet. Different transmission orders of the *n* packets may result in different energy waste. Let the sizes of the packets to be transmitted be {5,4,4,3,2,2}, the battery capacity be 10, and the energy to be received in each period be 10. Assume that the sensor can only receive two periods of wireless energy. If the packets are transmitted in the order of 5, 3, 2, 4, 4, 2, then no energy overflows in the first two periods as shown in Figure 3a. If the packets are transmitted in the order of 5, 4, 4, 3, 2, 2, then 1 unit of energy overflows and one unit of energy is unused, as shown in Figure 3b. Except energy overflowing, unused energy should also be avoided. In Figure 3c, if the packets are transmitted in the order of 2, 2, 3, 4, 4, 5, then three units of energy overflowing and two units of unused energy occurs if the sensor will not receive wireless energy anymore after two charging periods. Different orders of transmission may result in different energy waste.

### 2.3. Problem Statement

Sensors are mainly used to transmit collected data or forward data from other sensors to a base station in a sensor network. A packet can be transmitted when there is enough energy or it needs to wait until the sensor receives enough energy. Assume there are *n* packets to be transmitted or forwarded. How to transmit as much amount of data as possible while consuming as little energy as possible and taking full advantage of the wireless receiving energy is a problem. Assume that the transmission rate is much higher than the low power wireless charging rate. Then, data can be transmitted immediately as long as there is enough energy.

Let the capacity of the battery equipped on a sensor be *C*. In each period, the sensor receives *E* units of wireless energy. Let C=mE, i.e., the battery is full after *m* periods of wireless charging. For simplicity, let *m* be an integer. In this paper, we assume that the battery is empty initially. Given *n* packets to be transmitted to the base station, packet *i* is of size $s_{i}$ which means it costs $s_{i}$ units of energy to transmit this packet. The object of our problem is to minimize energy waste, or, on the other side, to maximize the amount of transmitted data in the periodical wireless charging mechanism. As shown in Figure 3, different transmission orders result in different energy waste. A wise transmission order wastes little energy, which is a rare resource in sensors and transmits as much amount of data as possible.

We first consider the situation where the number of wireless charging periods *K* is given. The problem can be formalized as follows.

**Definition** **1.** Let the battery capacity be C, and the charging energy in each period be E, where C=mE. Given n packets, $S=\{s_{1},s_{2},\cdots ,s_{n}\}$, where $s_{i}$ is the size of packet i, the problem is to find a transmission order to maximize the amount of transmitted data in K periods of wireless charging.

This problem is the case that sensors stay within the range of wireless power source for a limited time, i.e., *K* charging periods. In such a case, the sensors need to transmit as much amount of data as possible in the limited charging time.

If the sensors can carry out wireless charging for a long enough time, we then consider another version of this problem, which is formalized as follows.

**Definition** **2.** Let the battery capacity be C, and charging energy in each period be E, where C=mE. Given n packets $S=\{s_{1},s_{2},\cdots ,s_{n}\},$ where $s_{i}$ is the size of packet i, the problem is to find a transmission order to minimize the number of wireless charging periods so that all the data can be transmitted.

## 3. Transmission in Limited *K* Periods

If the sensors stay in the range of wireless charging for a limited period of time, then the sensors may not receive enough energy to transmit all the data kept in the memory. Some data may have to wait to be transmitted in the future. In such a situation, we try to solve the problem to maximize the amount of data that could be transmitted in the limited *K* periods. We first consider a simple situation where C=E, that is, the energy received in each period equals battery capacity. A battery is fully charged after only one period of wireless charging. This is the case where energy should be used as soon as possible. We then study the problem with C=2E, where a battery can be filled in two periods of wireless charging. Finally, we discuss the general problem with C=mE, where a battery can be filled in *m* periods of wireless charging.

### 3.1. A Simple Problem with C=E

When C=E, the energy received in each period is the same as battery capacity. In our assumption, all the data will be transmitted to the base station and no data will be discarded, so we need to transmit as much data as possible in order to completely make use of the received wireless energy without energy waste. Note that, because all the data will be transmitted, it prefers to transmit more data, rather than more packets. In the example illustrated in Figure 3, 20 units of data will be transmitted in two periods if the transmitted order of the packets is as that in Figure 3a, while 19 units of data will be transmitted in two periods in Figure 3b, and only 15 units of data can be transmitted in two periods in Figure 3c. If we want to transmit as much data as possible, we need to charge the battery to as full as possible. It is better to leave no residual space in one period, which may result in energy overflowing in the next period.

This problem is NP-Complete that is a class of problems which are hard to solve . One instance of this problem can be represented as the Partition problem, which is NP-Complete. The Partition problem is to partition a set of *n* integers $\left\{a_{1},a_{2},\ldots ,a_{n}\right\}$ into two subsets $A_{1}$ and $A_{2}$ such that the sums of both subsets equal to each other, i.e., $\sum_{a\in A_{1}}a=\sum_{a\in A_{2}}a$.

**Figure 4:**
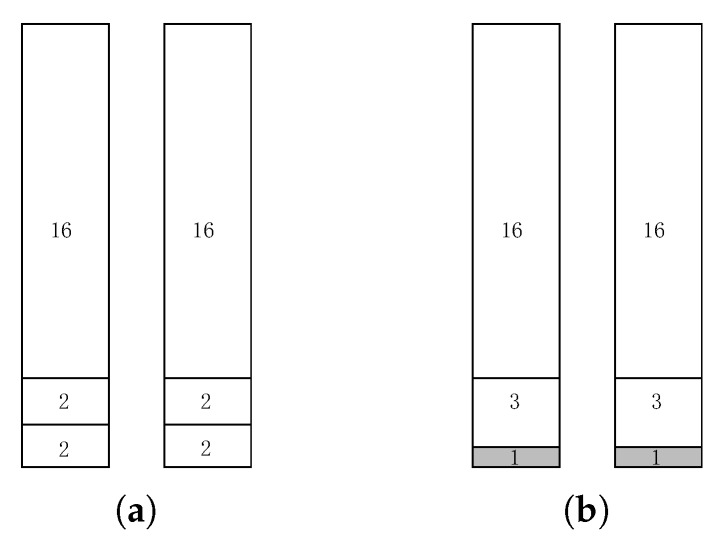
An example, (**a**) optimal solution; (**b**) larger packet first.

Throughout this paper, we use filling battery with packets to denote transmitting packets using one battery of energy, and filling period with packets to denote transmitting packets using one period of energy for simplicity. Intuitively, filling the battery with larger packets first would be a good solution. Figure 4 gives an example, S={16,16,3,3,2,2,2,2}, C=20, E=20 and K=2. It only wastes two units of data in this example as shown in Figure 4b. This method provides 2-approximation as shown in Theorem 1.

**Theorem** **1.** Filling battery with larger packet first is a 2-approximation algorithm.

**Proof.**  The method is described in Algorithm 1. Let OPT be an optimal solution that *K* periods of energy are all filled with packets, and no energy is wasted. Then, the amount of data that is transmitted in OPT is KC. We will prove the theorem by proving that the amount of transmitted data in Algorithm 1 is at least $\frac{K\cdot C}{2}$. For $k=1,2,\ldots ,\lfloor \frac{K}{2}\rfloor$, it is easy to find out that the total amount of data filled in battery 2k−1 and 2k is more than *C*, that is,

(1) $$\begin{array}{c} \sum_{i:trans(i)=2k-1}s_{i}+\sum_{i:trans(i)=2k}s_{i}>C. \end{array}$$

This is easy to understand because, if the total amount of data filled in battery 2k−1 and 2k is less than *C*, then it does not have to fill the data into two batteries, and it only needs to fill the data in one battery (2k−1 or 2k). Summing Inequality (1) up, we have

(2) $$\begin{array}{c} \sum_{i:trans\left(i\right)=\{1,\;2,\;\cdots ,\;2\left\lfloor \frac{K}{2}\right\rfloor \}}s_{i}>\left\lfloor \frac{K}{2}\right\rfloor C. \end{array}$$

If *K* is even, we have

(3) $$\begin{array}{c} \sum_{i:assign(i)=\{1,\;2,\;\cdots ,\;K\}}s_{i}>\frac{K}{2}C. \end{array}$$

Otherwise, if *K* is odd, we only need to consider whether half of the last battery is filled. Because we fill the packets in decreasing order, we always select a packet with a larger size first. Assuming less than half of the last battery is filled, and letting the size of the last packet packed in this battery be $s_{x}$, there must exist a packet with size $s_{y}<s_{x}\leq \frac{C}{2}$, and we can fill this packet into this battery, which contradicts the fact that $s_{x}$ is the last packet packed in this battery. Thus, at least half of the last battery is filled. Therefore, when *K* is odd, we still have

(4) $$\begin{array}{c} \sum_{i:trans(i)=\{1,\;2,\;\cdots ,\;K\}}s_{i}>\frac{K}{2}C. \end{array}$$

This completes the proof. ☐

A tight example is given as S={5+ϵ,5+ϵ,5+ϵ,5,5,5,5,5,5}, C=10, E=10 and K=3. In OPT, we can fill the six packets with size 5 into the three batteries, and the total amount of data is 30. However, in Algorithm 1, only the three packets with size 5+ϵ will be filled in the three batteries, and the total amount of transmitted data is 15+3ϵ.

**Algorithm 1:** FillDecreasing (*S*) Input: $S=\{s_{1},s_{2},\cdots ,s_{n}\}$ and *K*, assuming $\forall s_{i}\leq C$
Output: Transmit a subset $S^{\prime }\subset S$ of packets to maximize the total amount of data.
 1: Sort *S* in non-increasing order. Let the ordered sequence be $s_{1}\geq s_{2}\geq \ldots \geq s_{n}$;
 2: **for** j=1 to *K* **do**
 3:  e=C;
 4:  **for** *i* = 1 to *n* **do**
 5:   **if** $e\geq s_{i}$ **then**
 6:    trans(i)=j; //Transmitting packet *i* in battery *j*
 7:    $e=e-s_{i}$;
 8:   **end if**
 9:  **end for**
10: **end for**

Readers may wonder whether packets should be transmitted in an increasing order. We use a counterexample to show that transmitting packets in an increasing order of their sizes may still have a bad result. Given S={ϵ,10}, C=10, E=10 and K=1. In OPT, it transmits the packet with size 10, which will deplete the energy, while if transmitting packets in an increasing order of their sizes, only ϵ units of energy is used, but 10−ϵ units of energy will be left unused. This example shows that transmitting packets in an increasing order cannot bound the energy utilization rate.

**Lemma** **1.** When K=1, filling batteries with packets in decreasing order is a 2-approximation algorithm, but filling batteries with packets in another order is unbounded.

This can be shown from two examples. In the case S={5+ϵ,5,5}, C=10, E=10 and K=1, filling batteries in decreasing order results in $\lim_{\epsilon \to 0}\frac{10}{5+\epsilon }=2$. In the previously mentioned example, S={ϵ,10}, C=10, E=10 and K=1, filling batteries in increasing order results in $\lim_{\epsilon \to 0}\frac{10}{\epsilon }=+\infty$.

### 3.2. Problem with C=2E

Next, we consider the case where C=2E. Under the assumption that the transmission rate is much higher than the low power wireless charging rate, data can be transmitted immediately as long as there is enough energy.

**Lemma** **2.** The remaining energy in battery x is always less than E after transmitting a packet (i.e., x<E).

If the remaining energy is more than *E*, it means that it receives at least one period of energy after transmitting a packet. This lemma also indicates that the energy overflowing is always less than *E* after transmitting any packet.

**Lemma** **3.** When packet size $s_{i}\leq E$, transmitting this packet does not result in energy overflowing.

If $s_{i}\leq E$, then it waits at most one period to receive enough energy. Let the remaining energy in the battery be *x*, $x<s_{i}$ and $s_{i}\leq E$, and, after one period of wireless charging, the energy in the battery is x+E. Then, we have $s_{i}\leq x+E$, which means that the energy is enough for transmitting packet of size $s_{i}$ after one period of wireless charging.

This lemma also indicates that, if all packet sizes are less than *E*, then only the last period of energy may not be completely used. It means that the energy waste is at most *E* when transmitting any number of packets with a size less than *E*.

**Corollary** **1.** Energy overflowing occurs only when transmitting packet of size $s_{i}>E$.

From Lemma 3, we can see that, except for the energy waste in the last period of wireless charging, other energy waste occurs only when packet size $s_{i}>E$.

It seems that transmitting shorter packets with higher priority would be a good solution from Lemma 3. When all packets are of a size less than *E*, transmitting shorter packets first is indeed a good choice. It may waste only the last period of energy at most.

However, there exists a bad case in OPT, where the total KE units of energy are completely used, but transmitting shorter packets with higher priority may get a bad result, and leave a lot of energy unused.

We use an example to show such a case. Letting S={1,1,9,9}, C=10, E=5 and K=4, it can be found that, if we transmit the packets in the sequence of 1, 9, 1, 9, the energy is fully used. However, if we transmit shorter packet with higher priority, then, in the first period, it can transmit the two packets with total size of 2. The remaining energy in the battery is 3. It needs to wait for two periods to receive enough energy to transmit one packet with size 9, thus three units of energy overflow, and one unit of energy remains after the transmission. In the last period, it receives energy of 5, while the total six units of energy are not enough to transmit the last packet with size 9. Therefore, we can find that three units of energy overflow and six units of energy are unused, and the total energy waste is 9.

When we use the example of S={ϵ,ϵ,10−ϵ,10−ϵ}, C=10, E=5 and K=4, we can find that the total wasted energy is 10−ϵ, which means the ratio bound of transmitting shorter packets with higher priority is at least 2. If K=2, then only 2ϵ units of energy are used while 10−2ϵ energy are wasted. In this case, the approximation ratio is unbounded since $\lim_{\epsilon \to 0}\frac{C}{2\epsilon }=+\infty$.

Transmitting larger packet with higher priority is a 2-approximation algorithm that can be easily obtained from Theorem 1 by receiving two periods of energy at one time.

**Theorem** **2.** The energy utilization of Algorithm 2 is at least $\frac{3}{4}\left(OPT-E\right)$ .

**Proof.**  Let OPT be the energy used in the optimal solution. The perfect solution is that OPT=KE.

When all the packets are shorter than *E*, transmitting these packets will not waste energy unless it is the last packet that can be transmitted as described in Lemma 3. Therefore, even if the last period of energy is not completely used, the energy utilization is $OPT-E>\frac{3}{4}\left(OPT-E\right)$.

We now consider the situation where there are packets longer than *E*. Because the energy overflowing happens only when transmitting packet with size $s_{i}>E$. So the worst case is that energy overflowing happens as many times as possible. Let the energy left in the battery be *x*. When transmitting a packet with size $s_{i}>x+E$, there are *x* units of energy overflow. After transmitting this packet, the energy left in the battery is $y=2E-s_{i}<E-x$. When transmitting a packet with $s_{i+1}>y+E$, there are *y* units of energy overflow. It can be found that there are totally x+y<E energy overflows when transmitting these two packets. Therefore, it indicates that transmitting two larger packets continuously may result in at most *E* units of energy overflow. That is, there are at most *E* units of energy overflow in 4 periods of wireless charging, i.e., the ratio is $\frac{3}{4}$. In the first $4\lfloor \frac{K}{4}\rfloor$ periods, there are at most $\lfloor \frac{K}{4}\rfloor E$ units of energy overflow. The energy utilization in the first $4\lfloor \frac{K}{4}\rfloor$ periods is $\frac{3}{4}\cdot 4\left\lfloor \frac{K}{4}\right\rfloor E$. Because (Kmod4)≤3, we need to consider the following three cases.

Case 1: (Kmod4)=1, i.e., $OPT=(4\left\lfloor \frac{K}{4}\right\rfloor +1)E$. Even if the whole last period of energy is unused, the energy utilization is $\frac{3}{4}\cdot 4\left\lfloor \frac{K}{4}\right\rfloor E\geq \frac{3}{4}\left(OPT-E\right)$.

Case 2: (Kmod4)=2, i.e., $OPT=(4\left\lfloor \frac{K}{4}\right\rfloor +2)E$. Assume x>E units of energy in the last two periods are unused. It means that all the remaining packets have size larger than *x*. It contradicts the fact that the larger packet whose size is larger than *x* should be transmitted first. Therefore, in this case, at most *E* units of energy may be unused. This derives that the energy utilization is at least $\frac{3}{4}\left(OPT-E\right)$.

Case 3: (Kmod4)=3, i.e., $OPT=(4\left\lfloor \frac{K}{4}\right\rfloor +3)E$. Assume $x>\frac{E}{2}+E$ units of energy in the last three periods are unused. It means that all the remaining packets have a size larger than $x>\frac{3}{2}E$. It contradicts the fact that the larger packet whose size is larger than *x* should be transmitted first. Therefore, in this case, at most $\frac{3}{2}E$ units of energy may be unused. This derives that the energy utilization is at least $\frac{3}{4}\left(OPT-E\right)$.

This completes the proof. ☐

**Algorithm 2:** LargerFirst (*S*) Input: $S=\{s_{1},s_{2},\cdots ,s_{n}\}$, *C*, *E* and *K*, assuming $\forall s_{i}\leq C$
Output: Transmit a subset $S^{\prime }\subset S$ of packets to maximize the total amount of data.
 1: Sort *S* in non-increasing order, and let the ordered sequence be $s_{1}\geq s_{2}\geq \ldots \geq s_{n}$;
 2: e=0; k=0
 3: **for** *i* = 1 to *n* **do**
 4:  **while** $e<s_{i}$ and k<K **do**
 5:   e=e+E; //Wireless charging
 6:   k=k+1;
 7:  **end while**
 8:  **if** e>C **then**
 9:   e=C; //Energy overflows
10:  **end if**
11:  **if** $e<s_{i}$ **then**
12:   exit;
13:  **end if**
14:  Transmit packet *i*;
15:  $e=e-s_{i}$;
16: **end for**

After transmitting a larger packet, the remaining energy can be dedicated to transmitting some shorter packets. Since the more energy remains in the battery, the more energy may overflow. Such an improvement is described in Algorithm 3. In many cases, Algorithm 3 can improve energy utilization. However, in the worst case, it has the same energy utilization as Algorithm 2.

**Algorithm 3:** LargerFirstImprove (*S*) Input: $S=\{s_{1},s_{2},\cdots ,s_{n}\}$, *C*, *E* and *K*, assuming $\forall s_{i}\leq C$
Output: Transmit a subset $S^{\prime }\subset S$ of packets to maximize the total amount of data.
 1: Sort *S* in non-increasing order, and let the ordered sequence be $s_{1}\geq s_{2}\geq \ldots \geq s_{n}$;
 2: Append them into list *L* in non-increasing order;
 3: e=0; k=0
 4: **while** *L* is not empty **do**
 5:  Get the first packet *s* in list *L*.
 6:  **while** e<s and k<K **do**
 7:   e=e+E; //Wireless charging
 8:   k=k+1;
 9:  **end while**
10:  **if** e>C **then**
11:   e=C; //Energy overflows
12:  **end if**
13:  **for** $s^{\prime }\in L$ **do**
14:   Transmit packet $s^{\prime }$; // Transmit as more data as possible
15:   $e=e-s^{\prime }$;
16:   Remove $s^{\prime }$ from *L*;
17:  **end for**
18:  **if** k≥K **then**
19:   exit;
20:  **end if**
21: **end while**

### 3.3. The General Problem with C=mE

In the general case where C=mE, we can get a similar result as the problem with C=2E.

**Lemma** **4.** When packet size $s_{i}\leq \left(m-1\right)E$, transmitting this packet does not result in energy overflowing.

**Corollary** **2.** Energy overflowing happens only when transmitting packet of size $s_{i}>\left(m-1\right)E$.

One can use Algorithm 2 and Algorithm 3 to solve the problem with C=mE. The energy utilization is shown in Theorem 3.

**Theorem** **3.** The energy utilization of Algorithm 2 is at least $\left(1-\frac{1}{2m}\right)\left(OPT-E\right)$.

**Proof.**  Let OPT be the energy used in the optimal solution. The perfect solution is that OPT=KE.

Similar to the proof of Theorem 2, when all the packets are shorter than (m−1)E, transmitting these packets does not result in energy overflowing. Therefore, even if the energy charged in the last period is not used, the energy utilization $OPT-E>\left(1-\frac{1}{2m}\right)\left(OPT-E\right)$.

We now consider the situation where there are packets longer than (m−1)E. Because the energy overflowing happens only when transmitting packet with size $s_{i}>\left(m-1\right)E$, the worst case is that energy overflow happens as many times as possible. Let the energy left in the battery be *x*. When transmitting a packet with size $s_{i}>x+\left(m-1\right)E$, *x* units of energy overflow. After transmitting this packet, the energy left in the battery is $y=mE-s_{i}<E-x$. When transmitting a packet with $s_{i+1}>y+\left(m-1\right)E$, then *y* units of energy overflow. It can be found that totally x+y<E energy overflows when transmitting these two packets. Therefore, it indicates that transmitting two larger packets continuously may result in at most *E* units of energy overflow. That is, at most *E* units of energy overflow in 2m periods of wireless charging, i.e., the ratio is $\frac{2m-1}{2m}=1-\frac{1}{2m}$. In the first $2m\lfloor \frac{K}{2m}\rfloor$ periods, at most $\lfloor \frac{K}{2m}\rfloor E$ units of energy overflow. The energy utilization in the first $2m\lfloor \frac{K}{2m}\rfloor$ periods is $\left(1-\frac{1}{2m}\right)\cdot 2m\left\lfloor \frac{K}{2m}\right\rfloor E$. Similar to the analysis in Theorem 2, we consider the last (Kmod2m)≤2m−1 periods.

Similar to the analysis in Theorem 2, if (Kmod2m)≤m, at most *E* units of energy are unused. This derives that the energy utilization is at least $\left(1-\frac{1}{2m}\right)\left(OPT-E\right)$.

If m<(Kmod2m)≤2m−1, at most $\frac{E}{2}+E$ units of energy are unused. This derives that the energy utilization is at least $\left(1-\frac{1}{2m}\right)\left(OPT-E\right)$.

This completes the proof. ☐

## 4. Transmission in an Unlimited Number of Periods

If sensors stay in the range of wireless charging for a long enough time, then all the data can be transmitted to the base station. In such a situation, we try to solve the problem to minimize the number of the used periods. This is reasonable, since it needs to store as much energy as possible to transmit packets arriving in the future. Similar to the last section, we first consider a simple situation where C=E, i.e., the energy received in each period equals the battery capacity. We then study the problem with C=2E, where the battery can be filled in two periods of wireless charging. Finally, we discuss the general problem with C=mE, where the battery can be filled in *m* periods of wireless charging.

### 4.1. A Simple Problem with C=E

When C=E, the problem is exactly the well known Binpacking problem [20]. The Binpacking problem is defined as follows. Given *n* items with sizes $s_{1}$, $s_{2}$, …, $s_{n}$, and *n* bins with capacity *C*, the problem is to pack the *n* items into the bins such that the number of used bins is minimum.

The Binpacking problem is NP-Complete, and some approximation algorithms have been proposed. We show some existing theorems to better understand this problem.

**Theorem** **4.** There is no α-approximation algorithm with $\alpha <\frac{3}{2}$ for the Binpacking problem unless P = NP.

This theorem can be derived immediately from the Partition problem. The Partition problem is to partition a set of *n* integers $\left\{a_{1},a_{2},\ldots ,a_{n}\right\}$ into two subsets $A_{1}$ and $A_{2}$ such that the sums of both subsets are equal, i.e., $\sum_{a\in A_{1}}a=\sum_{a\in A_{2}}a$. The Partition problem is also NP-Complete, and it is easy to find out that the Partition problem is a simple instance of the Binpacking problem. If the Binpacking problem has an approximation algorithm with ratio bound less than $\frac{3}{2}$, then we can use this algorithm to solve the Partition problem, and it will derive an optimal result with less than three subsets (bins), which is exactly two subsets (bins). The approximation algorithm is a polynomial time algorithm, which contradicts the fact that the Partition problem is NP-Complete.

**Theorem** **5.** The FirstFitDecreasing algorithm is $\frac{3}{2}$-approximation for the Binpacking problem.

The FirstFitDecreasing algorithm is described as follows. Sort the items such that $s_{1}\geq s_{2}\geq \ldots \geq s_{n}$. Initially, all the bins are empty and we start with bin k=0 and item i=1. Consider all bins j=1,…,k and place item *i* in the first bin that has sufficient residual capacity. If there is no such bin, increase *k* and repeat until item *n* is assigned. The FirstFitDecreasing algorithm is the best approximation algorithm currently for the Binpacking problem.

Regarding bins as the periods of energy, our problem is the same as the Binpacking problem, and the FirstFitDecreasing algorithm can generate $\frac{3}{2}$-approximation.

### 4.2. Problem with C=2E

A $\frac{3}{2}$-approximation algorithm can be easily derived by receiving two periods of energy at one time and using Algorithm 4. We show a better algorithm in Algorithm 5.

**Algorithm 4:** FirstFitDecreasing (*S*) Input: $S=\{s_{1},s_{2},\cdots ,s_{n}\}$, assuming $\forall s_{i}\leq C$
Output: The assignment of *S* and *k*
 1: Sort *S* in non-increasing order, and let the ordered sequence be $s_{1}\geq s_{2}\geq \ldots \geq s_{n}$;
 2: k=0;
 3: **for** *i* = 1 to *n* **do**
 4:  **for** j=1 to *k* **do**
 5:   **if** $Bin\left[j\right]\geq s_{i}$ **then**
 6:    assign(i)=j; //assign item *i* to *j*, corresponding to transmit packet *i* in period *j*.
 7:    $Bin\left[j\right]=Bin\left[j\right]-s_{i}$;
 8:   **end if**
 9:  **end for**
10:  **if** j>k **then**
11:   k=k+1;
12:   Bin[k]=C;
13:   assign(i)=k;
14:   $Bin\left[k\right]=Bin\left[k\right]-s_{i}$;
15:  **end if**
16: **end for**

**Theorem** **6.** Algorithm 5 uses no more than $\frac{4}{3}OPT+1$ periods of energy.

**Proof.**  Let OPT be the number of periods of energy used in an optimal solution. When all the packets are shorter than *E*, transmitting these packets will not waste energy unless it is the last packet that can be transmitted as described in Lemma 3. Therefore, even if the energy charged in the last period is not used, it uses at most $OPT+1\leq \frac{4}{3}OPT+1$ periods of energy.

We now consider the situation where there are packets longer than *E*. Because the energy overflowing happens only when transmitting a packet whose size is $s_{i}>E$, the worst case is that energy overflow happens as many times as possible. The previous proof shows that transmitting two larger packets continuously may result in at most *E* units of energy overflow. Assume there are *N* packets that result in energy overflowing. Letting these packets be a set *S*, i.e., |S|=N, and the remaining packets be $S^{\prime }$, then $OPT\cdot E=\sum_{s\in S}s+\sum_{s\in S^{\prime }}s$.

If *N* is even, then at most $\frac{1}{2}NE$ units of energy overflow, i.e., at most $\frac{4}{3}\sum_{s\in S}s$ units of energy are used to transmit these *N* packets. The packets in $S^{\prime }$ will not result in energy overflowing, but the last period of energy may not be used completely. Thus, transmitting larger packets first will use at most $\frac{4}{3}\sum_{s\in S}s+\sum_{s\in S^{\prime }}s+E\leq \left(\frac{4}{3}OPT+1\right)E$ units of energy.

If *N* is odd, let the first N−1 packets in *S* be $S_{A}$, and let the last packet in *S* be $s_{z}$. Obviously, $S_{A}=N-1$ is even. Transmitting these packets uses at most (N−1)·2E units of energy, and at most $\frac{N-1}{2}E$ units of energy overflow. That is, at most $\frac{4}{3}\sum_{s\in S_{A}}s$ units of energy are used to transmit the N−1 packets. Note that, because the battery is empty initially, the global first transmitted packet $s_{first}$ will not result in energy overflow (remember that $s_{first}\in S^{\prime }$). Transmitting these two packets $s_{first}$ and $s_{z}$ will use at most $\frac{4}{3}\left(s_{first}+s_{z}\right)$ units of energy. Then, it derives $\frac{4}{3}\left(\sum_{s\in S_{A}}s+s_{z}+s_{first}\right)+\left(\sum_{s\in S^{\prime }}s-s_{first}\right)+E\leq \left(\frac{4}{3}OPT+1\right)E$.

This completes the proof. ☐

Similar to the previous section, after transmitting a larger packet, the remaining energy can be dedicated to transmitting some shorter packets. Such an improvement is described in Algorithm 6. In many cases, Algorithm 6 can improve energy utilization. However, in the worst case, it has the same energy utilization as Algorithm 5.

### 4.3. The General Problem with C=mE

**Theorem** **7.** Algorithm 5 uses no more than $\frac{2m}{2m-1}OPT+1$ periods of energy.

The proof is similar to that of the problem with C=2E, and it is neglected.

**Algorithm 5:** LargerFirst2 (*S*) Input: $S=\{s_{1},s_{2},\cdots ,s_{n}\}$, *C*, *E* and *K*, assuming $\forall s_{i}\leq C$
Output: Transmit a subset $S^{\prime }\subset S$ of packets to maximize the total amount of data.
 1: Sort *S* in non-increasing order, and let the ordered sequence be $s_{1}\geq s_{2}\geq \ldots \geq s_{n}$;
 2: e=0;
 3: **for** *i* = 1 to *n* **do**
 4:  **while** $e<s_{i}$ **do**
 5:   e=e+E; //Wireless charging
 6:  **end while**
 7:  **if** e>C **then**
 8:   e=C; //Energy overflows
 9:  **end if**
10:  Transmit packet *i*;
11:  $e=e-s_{i}$;
12: **end for**

**Algorithm 6:** LargerFirstImprove2 (*S*) Input: $S=\{s_{1},s_{2},\cdots ,s_{n}\}$, *C*, *E* and *K*, assuming $\forall s_{i}\leq C$
Output: Transmit a subset $S^{\prime }\subset S$ of packets to maximize the total amount of data.
 1: Sort *S* in non-increasing order, and let the ordered sequence be $s_{1}\geq s_{2}\geq \ldots \geq s_{n}$;
 2: Append them into list *L* in non-increasing order;
 3: e=0;
 4: **while** *L* is not empty **do**
 5:  Get the first packet *s* in list *L*.
 6:  **while** e<s **do**
 7:   e=e+E; //Wireless charging
 8:  **end while**
 9:  **if** e>C **then**
10:   e=C; //Energy overflows
11:  **end if**
12:  **for** $s^{\prime }\in L$ **do**
13:   Transmit packet $s^{\prime }$; // Transmit as much data as possible
14:   $e=e-s^{\prime }$;
15:   Remove $s^{\prime }$ from *L*;
16:  **end for**
17: **end while**

## 5. Simulations

The simulation compares the larger first algorithm used in this paper to a greedy approximation algorithm, which transmits the first fit packet first. For ease of comparison, let the larger first decreasing algorithm analysed in this paper be LF, and the first fit algorithm be FF.

**Figure 5:**
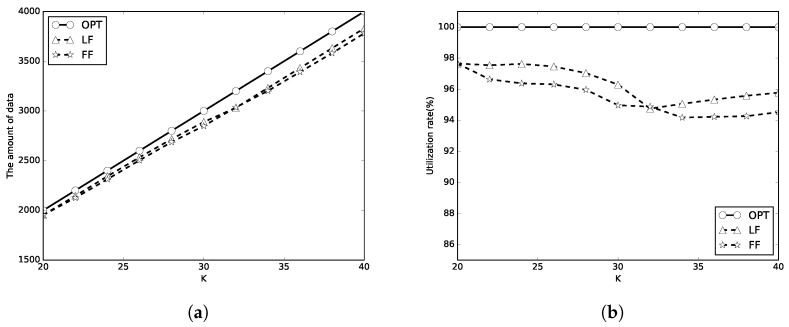
C=E. (**a**) the amount of data; (**b**) energy utilization rate.

**Figure 6:**
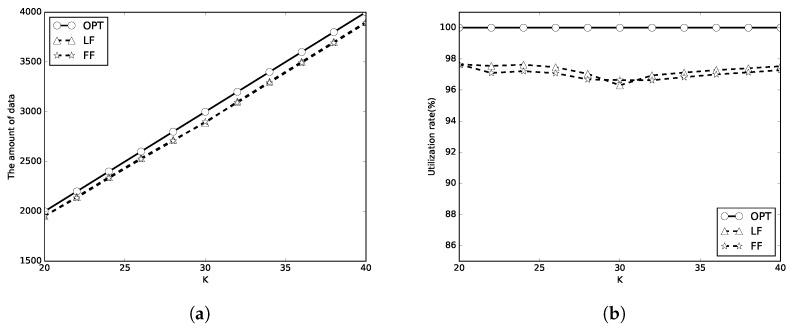
C=2E. (**a**) the amount of data; (**b**) energy utilization rate.

**Figure 7:**
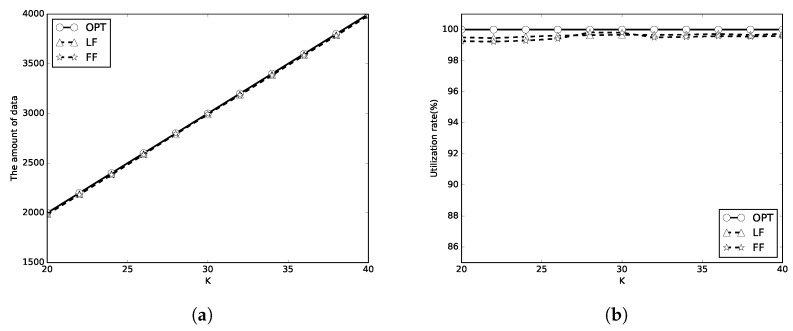
C=10E. (**a**) the amount of data; (**b**) energy utilization rate.

In the first set of simulations, we set C=100 and m={1,2,10}. We first evaluate the performance of the algorithms with limited *K* periods of wireless charging. *K* is set to [20, 40], the number of packets is 100, and the packet sizes are random numbers in [1, 100]. The simulation results are shown in Figure 5, Figure 6 and Figure 7. In these figures, the optimal, LF, FF results are representsed as circle, triangle and star lines, respectively. A better algorithm will transmit more amount of data, and has a higher utilization rate. It can be be found that the amount of transmitted data by LF algorithm used in this paper and FF algorithm is close to that of OPT in Figure 5a, Figure 6a and Figure 7a. The energy utilization rate of LF and FF algorithm is also close to that of OPT (which is 100%) as shown in Figure 5b, Figure 6b and Figure 7b. It means that the proposed algorithms almost completely use the total *K* periods of energy. When *m* is larger, the energy utilization rate is better. When *m* is larger, the energy waste is less. The LF algorithm outperforms the FF algorithm in most of the simulation cases.

**Figure 8:**
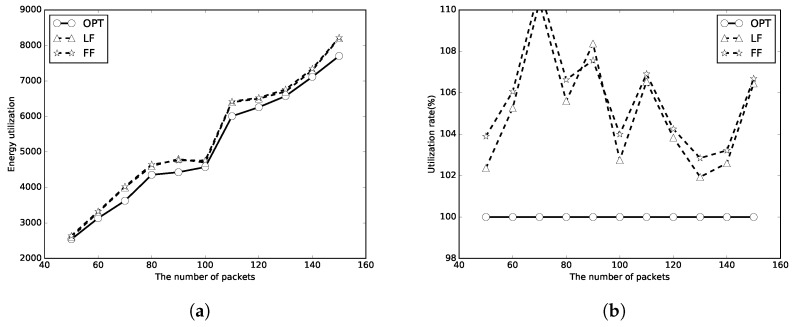
C=E. (**a**) energy utilization; (**b**) energy utilization ratio.

**Figure 9:**
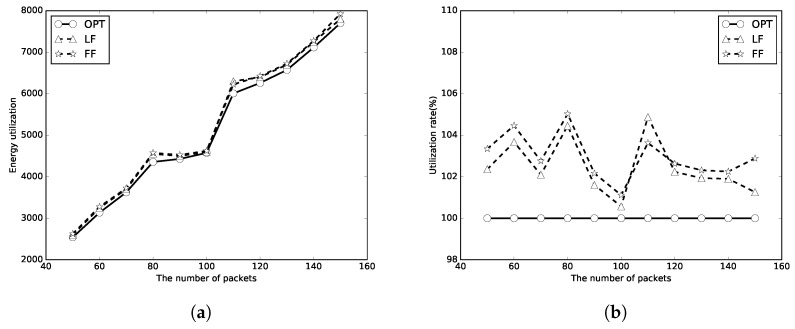
C=2E. (**a**) energy utilization; (**b**) energy utilization ratio.

**Figure 10:**
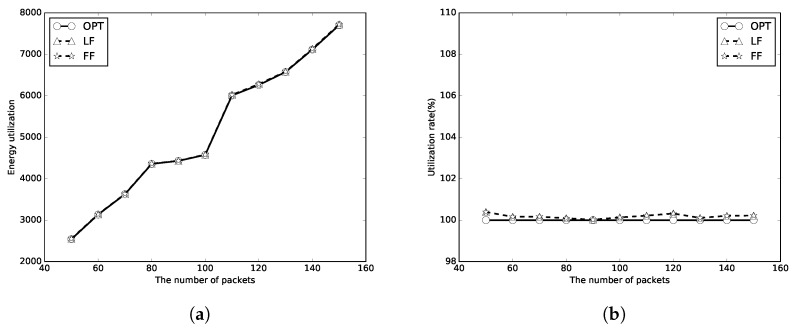
C=10E. (**a**) energy utilization; (**b**) energy utilization ratio.

We next evaluate the performance of the proposed algorithms with unlimited numbers of periods of wireless charging. The number of packets *n* is set to [50, 150], and the packet sizes are random numbers in [1, 100]. Because there is enough energy to transmit all the packets, it thus uses the energy utilizations to represent the performances of the algorithms. A better algorithm will use less energy, which means less energy waste and has a higher utilization rate. Again, the optimal, LF, FF results are represented as circle, triangle and star lines, respectively. It can be found that the energy used of LF and FF algorithms is close to that of OPT in Figure 8a, Figure 9a and Figure 10a. The energy utilization rate is also close to that of OPT (which is 100%) as shown in Figure 8b, Figure 9b and Figure 10b. It indicates that the algorithms use not too much more energy to transmit all the packets than OPT. As *m* increases, less energy will be used to transmit all the packets. The LF algorithm has a better performance than that of the FF algorithm in most of the simulation cases.

## 6. Related Work

Thermal effect on the tissues is the most important issues in biosensors. Even the temperature effect rised by wireless communication should be considered. Existing works usually limit the radio’s transmission power to reduce the temperature increasing and use the traffic control algorithms and to avoid routing from high temperature zones [21]. TARA (Thermal Aware Routing Algorithm) routes data through low temperature zones [11]. LTR (Least Temperature Routing) and ALTR (Adaptive Least Temperature Routing) reduce the irrelevant loops and hops by maintaining a list of recently visited nodes [22]. LTRT (Least Total Route Temperature) is a combination of short path routing and LTR to minimum temperature routes [23]. HPR considers the delay-sensitive situation such as medical monitoring [24], and RAIN studies the problem in networks of homogenous and Id-less biomedical sensor nodes [25]. This paper considers the thermal effect resulted by wireless charging.

Wireless charging can result in more serious thermal effects [26,27,28]. Wireless energy transfer can result in thermal effects on tissues is stated in many works [11,12,13,14,15,16,17,29]. The temperature of a wireless power receiver will rise as the energy is being transferred. Tang and Movassaghi et al. have shown that exposure to SAR of 8 W/kg for 15 min will result in severe tissue damage [11,29]. The methods used to eliminate the thermal effect include designing a cooling system, constraining the charging time or reducing the amount of workload so as to save the energy usage. This paper employs a periodical wireless charging mechanism so as to meet the thermal constraint and tries to transmit as much data as possible.

The NP-Completeness of the problem in this paper can be reduced from the Binpacking problem [20]. Thus, it is unlikely to design a polynomial time algorithm to solve the problem nowadays. People usually try to solve the problem with approximation algorithm. A greedy approximation algorithm that places the items in the first bin in arbitrary order can derive a 2-approximation factor [30]. The FirstFitDecreasing algorithm places the items into the first bins in decreasing order by their sizes. It will use no more than 11/9OPT+1 bins. Dosa et al. proved that the bound 11/9OPT+6/9 for FirstFitDecreasing is tight [31]. Dosa and Sgall also gave a tight upper bound for the first-fit strategy, which uses more than 17/10OPT bins for any situation [32]. The problem studied in this paper is different from the Binpacking problem in that the battery would be full only after *m* charging periods. Two cases are considered in this paper: one is that the charging time is limited and the other is that the charging time is long enough, and the objective of the problem of the first case is different from the Binpacking problem.

## 7. Conclusions

This paper proposes a class of algorithms to solve the problem of maximizing energy utilization in sensors with periodical wireless charging situations that take into account the constraint of wireless charging. Two types of problems are studied. The first is that there are only limited *K* periods of energy, while the second is that there are enough periods of energy to transmit the packets. For the first problem, because there is not enough energy, the objective is to transmit as much data as possible, which represents maximizing the energy utilization. Three cases where C=E, C=2E and C=mE correspond to the battery will be full after receiving 1, 2, *m* periods of wireless energy, respectively, are studied. We use the method of transmitting larger packets first and prove that the energy utilization of proposed algorithm is at least $\frac{1}{2}OPT$, $\frac{3}{4}\left(OPT-E\right)$, and $\left(1-\frac{1}{2m}\right)\left(OPT-E\right)$ for the three cases, respectively. For the second problem where there is enough energy, the objective is to transmit all the packets with the minimum amount of energy—again, three cases where C=E, C=2E and C=mE are studied. It is proved that it will use no more than $\frac{2m}{2m-1}OPT+1$ energy to transmit all the packets compared to the optimal solution for the general problem. The simulation results confirm the theoretical analysis and show that the performance is much better than that of the worst case in the theoretical analysis. It also indicates that the larger packet first algorithm used in this paper outperforms that of the first fit packet algorithm in the simulations. Therefore, algorithms used in this paper not only have a good theoretical performance such as the one that we analyzed, but also have a good simulation performance.

## References

[1] Tang Q., Tummala N., Gupta S., Schwiebert L. (2005). TARA: Thermal-Aware Routing Algorithm for Implanted Sensor Networks. Distributed Computing in Sensor Systems.

[2] Guo T., Zhang L., Liu W., Zhou Z. A novel solution to power problems in implanted biosensor networks. Proceedings of the 28th Annual International Conference of the IEEE on Engineering in Medicine and Biology Society.

[3] Cheng S., Cai Z., Li J. (2015). Curve Query Processing in Wireless Sensor Networks. IEEE Trans. Veh. Technol..

[4] Olivo J., Carrara S., De Micheli G. (2011). Energy Harvesting and Remote Powering for Implantable Biosensors. IEEE Sens. J..

[5] Schatz D., Hartford J. (2013). Wireless Power for Medical Devices. http://www.mddionline.com/article/wireless-power-medical-devices.

[6] Kim A., Maleki T., Ziaie B. A novel electromechanical interrogation scheme for implant able passive transponders. Proceedings of the IEEE 25th International Conference on Micro Electro Mechanical Systems.

[7] Katic J. (2015). Efficient Energy Harvesting Interface for Implantable Biosensors.

[8] Antonacci T. (2012). Cooling down Your Wireless Power Receiver. http://www.edn.com/design/power-management/4391074/Cooling-Down-your-Wireless-Power-Receiver.

[9] Gravina R., Alinia P., Ghasemzadeh H., Fortino G. (2017). Multi-sensor fusion in body sensor networks: State-of-the-art and research challenges. Inf. Fusion.

[10] Ghamari M., Janko B., Sherratt R.S., Harwin W.S., Piechockic R., Soltanpur C. (2016). A Survey on Wireless Body Area Networks for eHealthcare Systems in Residential Environments. Sensors.

[11] Tang Q., Tummala N., Gupta S., Schwiebert L. (2005). Communication scheduling to minimize thermal effects of implanted biosensor networks in homogeneous tissue. IEEE Trans. Biomed. Eng..

[12] Hirata A., Ushio G., Shiozawa T. (2000). Calculation of Temperature Rises in the Human Eye Exposed to EM Waves in the ISM Frequency Bands. IEICE Trans. Commun..

[13] Schneider J., Lucke L., Wessels D., Schauer T. Impacts of Wireless Power on Medical Device Design Safety. Proceedings of the Design of Medical Devices Conference.

[14] Frei M., Jauchem J., Padilla J., Merritt J. (1989). Thermal and physiological responses of rats exposed to 2.45-GHz radiofrequency radiation: A comparison of E and H orientation. Radiat. Environ. Biophys..

[15] Thiele J.G.M., Dössel O. (2009). Thermal Heating of Human Tissue Induced by Electro-Magnetic Fields of Magnetic Resonance Imaging. Biomed. Tech. Biomed. Eng..

[16] Psenakova Z., Psenak V. (2005). Electromagnetic Heating of Human Tissue. Acta Mech. Slovaca.

[17] Zheng X., Cai Z., Li J., Gao H. (2017). A Study on Application-aware Scheduling in Wireless Networks. IEEE Trans. Mob. Comput..

[18] Hyperphysics (2012). Cooling of the Human Body. http://hyperphysics.phy-astr.gsu.edu/hbase/thermo/coobod.html.

[19] Nilsson A.L. (1987). Blood Flow, Temperature, and Heat Loss of Skin Exposed to Local Radiative and Convective Cooling. J. Investig. Dermatol..

[20] Korte B., Vygen J. (2006). Chapter Bin-Packing. Combinatorial Optimization: Theory and Algorithms.

[21] Movassaghi S., Abolhasan M., Lipman J. (2013). A Review of Routing Protocols in Wireless Body Area Networks. J. Netw..

[22] Bag A., Bassiouni M.A. Energy Efficient Thermal Aware Routing Algorithms for Embedded Biomedical Sensor Networks. Proceedings of the IEEE International Conference on Mobile Adhoc and Sensor Systems.

[23] Takahashi D., Xiao Y., Hu F., Chen J., Sun Y. (2008). Temperature-aware routing for telemedicine applications in embedded biomedical sensor networks. EURASIP J. Wirel. Commun. Netw..

[24] Bag A., Bassiouni M.A. (2008). Hotspot Preventing Routing algorithm for delay-sensitive applications of in vivo biomedical sensor networks. Inf. Fusion.

[25] Bag A., Bassiouni M.A. Routing algorithm for network of homogeneous and id-less biomedical sensor nodes (RAIN). Proceedings of the Sensors Applications Symposium (SAS 2008).

[26] Xiao L., Ping W., Niyato D., Dong I.K., Zhu H. (2016). Wireless Charging Technologies: Fundamentals, Standards, and Network Applications. IEEE Commun. Surv. Tutor..

[27] Dai H., Liu Y., Chen G., Wu X., He T., Liu A.X., Ma H. (2014). Safe Charging for Wireless Power Transfer. IEEE/ACM Trans. Netw..

[28] Olteanu M., Marincaş C., Rafiroiu D. (2012). Dangerous Temperature Increase from EM Radiation around Metallic Implants. Acta Electroteh..

[29] Movassaghi S., Abolhasan M., Lipman J., Smith D., Jamalipour A. (2014). Wireless Body Area Networks: A Survey. IEEE Commun. Surv. Tutor..

[30] Vazirani V.V. (2003). Chapter Bin-Packing. Approximation Algorithms.

[31] Dósa G., Li R., Han X., Tuza Z. (2013). Tight Absolute Bound for First Fit Decreasing Bin-packing: FFD(L)<=11/9OPT(L)+6/9. Theor. Comput. Sci..

[32] Dósa G., Sgall J. First Fit bin packing: A tight analysis. Proceedings of the International Symposium on Theoretical Aspects of Computer Science.

